# Clinical outcomes of screen-positive genome-wide cfDNA cases for trisomy 20: results from the global expanded NIPT Consortium

**DOI:** 10.1186/s13039-024-00677-1

**Published:** 2024-04-16

**Authors:** Erica Soster, Tamara Mossfield, Melody Menezes, Gloudi Agenbag, Marie-Line Dubois, Jean Gekas, Tristan Hardy, Kelly Loggenberg, on behalf of the Global Expanded NIPT Consortium

**Affiliations:** 1 Labcorp, 3400 Computer Drive, Westborough, MA 01581 USA; 2 Genea, Sydney, NSW Australia; 3 Monash IVF Genetics, Monash IVF Group, Richmond, VIC Australia; 4 Next Biosciences, Johannesburg, South Africa; 5 Laval University, Quebec City, QC Canada; 6 Department of Medical Genetics, CHU de Quebec Research and Mother and Child Center, University Hospital of Quebec, Laval University, Quebec City, QC Canada; 7 SA Pathology, Adelaide, SA 5000 Australia; 8 Monash IVF Group, Melbourne, Victoria Australia

**Keywords:** Noninvasive prenatal testing, Chromosome 20, Trisomy, Rare autosomal aneuploidy, Pregnancy outcome, Mosaicism, Genome-wide

## Abstract

Trisomy 20 has been shown to be one of the most frequent rare autosomal trisomies in patients that undergo genome-wide noninvasive prenatal testing. Here, we describe the clinical outcomes of cases that screened positive for trisomy 20 following prenatal genome-wide cell-free (cf.) DNA screening. These cases are part of a larger cohort of previously published cases. Members of the Global Expanded NIPT Consortium were invited to submit details on their cases with a single rare autosomal aneuploidy following genome-wide cfDNA screening for retrospective analysis. Clinical details including patient demographics, test indications, diagnostic testing, and obstetric pregnancy outcomes were collected. Genome-wide cfDNA screening was conducted following site-specific laboratory procedures. Cases which screened positive for trisomy 20 (*n* = 10) were reviewed. Clinical outcome information was available for 90% (9/10) of our screen-positive trisomy 20 cases; the case without diagnostic testing ended in a fetal demise. Of the nine cases with outcome information, one was found to have a mosaic partial duplication (duplication at 20p13), rather than a full trisomy 20. Only one case in the study cohort had placental testing; therefore, confined placental mosaicism could not be ruled out in most cases. Adverse pregnancy outcomes were seen in half of the cases, which could suggest the presence of underlying confined placental mosaicism or mosaic/full fetal trisomy 20. Based on our limited series, the likelihood of true fetal aneuploidy is low but pregnancies may be at increased risk for adverse obstetric outcomes and may benefit from additional surveillance.

## Introduction

It has been over a decade since the clinical introduction of cell-free (cf.) DNA screening into the prenatal space, with numerous publications demonstrating the high accuracy of this screening test in the detection of common fetal trisomies (trisomies 21, 18, and 13) [1–4]. The use of cfDNA screening to test for the presence of common fetal trisomies is now recommended by many professional medical societies for all pregnant patients [5–9], however, professional societies note that more data is needed for cfDNA screening beyond the common trisomies. Genome-wide cfDNA screening can identify chromosomal aneuploidies that may impact a pregnancy beyond trisomies 21, 18, and 13, including rare autosomal aneuploidies (RAAs) and copy number variants. There has been an increasing number of studies detailing their clinical experience with genome-wide cfDNA screening in recent years [10–26]. Several of these studies have also detailed the adverse perinatal complications that can arise in some patients that screen-positive for RAAs or copy number variants following genome-wide cfDNA screening including preeclampsia, fetal growth restriction, intrauterine fetal demise, and preterm birth. However, the data specific to cases screening positive for trisomy 20 is relatively limited and thus this cohort attempts to contribute to the body of aneuploidy-specific literature. As more cases of rare aneuploidies by cfDNA are published and more information becomes available on the outcomes and phenotype for specific chromosomes, providers should theoretically be able to provide more tailored pregnancy management and counseling.

Studies have shown screen-positive rates for RAAs ranging from 0.12 to 1.1% [23, 27]. In our previous study looking at the impact of RAAs on pregnancy management and outcomes, we found that trisomy 20 was detected in 9.2% of patients with a screen-positive result for a RAA [12]. Another recent study noted that trisomy 20 was one of the most frequent rare autosomal trisomies in their patient cohort (11.5% of screen-positive cases) [18]. Trisomy 20 may be present in full or mosaic form in the fetus or placenta, although full fetal trisomy 20 typically results in an early pregnancy loss. The incidence of mosaic trisomy 20 on amniocentesis in a general pregnancy population is approximately 1 in 5000 [28]. A study looking at chromosomal abnormalities in products of conception following an early miscarriage found that trisomy 20 was present in about 1.2% of cases [29]. Chromosome 20 is also known to be imprinted and paternal uniparental disomy (UPD) phenotypes have been reported [30]. Mosaic trisomy 20 at amniocentesis is associated with a spectrum of outcomes that may not be correlated to the level of mosaicism [31–34]. Postnatal phenotypic features of mosaic trisomy 20 can include spinal abnormalities, hypotonia, lifelong constipation, sloped shoulders, and significant learning disabilities [34].

The objective of this study was to add to the body of evidence around prenatal screening for conditions beyond the common trisomies by describing the outcomes of a small cohort of patients that had a positive result for trisomy 20 following genome-wide cfDNA screening. These cases were previously published as part of a larger cohort of cases [12]. Diagnostic testing outcomes as well as pregnancy and birth outcomes for these cases are discussed.

## Methods

As noted above, the data in this study are based on a subset of previously published data [12]. As outlined in the prior publication, members of the Global Expanded NIPT Consortium were invited to submit details on their cases with a single RAA following genome-wide cfDNA screening for retrospective analysis. For this study, only cases that screened positive for the presence of trisomy 20 were included. All cases of trisomy 20 in the broader cohort were included in the dataset for the present study. Patient samples collected as part of routine cfDNA screening were included in this retrospective data analysis study, according to site-specific protocols and standards of care. Samples from both high-risk and low-risk pregnancy cohorts, along with singleton or twin samples, could be included in the study. Information including patient demographics, test referral indications, and information on human chorionic gonadotropin levels, pregnancy associated plasma protein levels, and nuchal translucency were collected if available. All data was de-identified before analysis was carried out.

Genome-wide cfDNA screening was carried out at each of the four sites according to their specific laboratory protocols; sites described in the original study that did not have trisomy 20 cases were excluded here [12]. Three of the four sites used the VeriSeq™ NIPT Solution v2 assay (Illumina, Inc.) [35], and one site used the TruSeq™ Nano 16 sample protocol (Illumina, Inc.) for cfDNA sequencing [36]. All four sites attempted to collect follow-up clinical information, including diagnostic testing outcomes and obstetric pregnancy outcomes, for each of their submitted cases. Concordance of cfDNA results with diagnostic outcomes were based on either fetal or placental testing. As in the previous publication, cases were considered concordant if they had either a full or mosaic trisomy 20 or UPD on chromosome 20.

## Results

Ten of the cases submitted by members of the Consortium screened positive for the presence of trisomy 20. All ten samples were from singleton pregnancies and were collected between 2017 and 2020. Maternal ages, gestational ages, and fetal fractions for each of the 10 patients are provided in Table 1. The median maternal age was 38.0 years, with a range of 32.0–47.0 years; in 90% of cases, the patient was over 35 years old at the time of testing. The median gestational age was 11.8 weeks, with a range of 10.3–15.3 weeks; most cases had testing in the first trimester. Fetal fractions ranged from 6 to 12%, with an average of 8% and a median of 9%. Two cases did not have fetal fractions available (non-interpretable fetal fraction results) as detailed in Mossfield et al. [12]. With regards to referral indications for cfDNA screening, six cases did not list a referral indication, three cases listed advanced maternal age, and one case listed primary screening (Table 1). The patient that listed primary screening as the referral indication had a beta human chorionic gonadotropin level of 0.41 multiple of the median, a pregnancy associated plasma protein level of 0.71 multiple of the median, and a nuchal translucency of 1.4 mm.

**Table 1:** Patient characteristics and referral indications (*n* = 10)

Case	Maternal age (years)	Gestational age (weeks + days)	Referral indication	Fetal fraction (%)
1	36	13 + 2	Advanced maternal age	12
2	32	10 + 2	Primary screening	6
3	39	12 + 0	Advanced maternal age	6
4	42	11 + 4	None specified	9
5	41	15 + 2	None specified	N/a
6	37	12 + 4	None specified	8
7	37	11 + 0	None specified	9
8	37	12 + 5	Advanced maternal age	6
9	39	10 + 3	None specified	N/a
10	47	10 + 5	None specified	9

N/a, not available

Diagnostic testing was carried out for 90% (9/10) of the screen-positive trisomy 20 cases (see Table 2); the one case without diagnostic testing ended in a fetal demise at 13 weeks of gestation. All nine cases underwent amniocentesis to determine fetal concordance, with one case (case #8) also having placental testing (postnatal) in addition to amniocentesis. All nine of the cases with testing on amniotic fluid had normal testing as indicated in Table 2. Only one case (case #2) had UPD testing, which returned a normal result, even though UPD testing is recommended for patients with a screen-positive cfDNA result on trisomy 20. Of the nine cases with diagnostic information, one was found to have a mosaic partial duplication (duplication at 20p13). The one case that had placental testing in addition to fetal testing was found to be discordant with the cfDNA screening result. The lack of placental testing in the other cases meant that confined placental mosaicism (CPM) could not be ruled out for those patients.

**Table 2:** Diagnostic testing outcomes and obstetric outcomes for study cohort

Case	Diagnostic testing results	Pregnancy complications	Pregnancy outcome	Category^a^
1	Amniocentesis: Normal microarray	Fetal macrosomia	Term livebirth	Discordant
2	Amniocentesis: Normal microarray and UPD studies	Preeclampsia	Term livebirth	Discordant with adverse outcome
3	Amniocentesis: Normal (unspecified testing)	Unavailable	Unavailable	Discordant with unknown outcome
4^b^	Amniocentesis: Normal microarray and FISH POC (cord): Normal (unspecified testing)	Multiple anomalies on ultrasound and autopsy	Elective termination	Discordant with adverse outcome
5^c^	Amniocentesis: Normal microarray	Fetal growth restriction, gestational diabetes; Emergency preterm birth	Livebirth	Discordant with adverse outcome
6^d^	Amniocentesis: Normal karyotype	Gestational diabetes; Preterm delivery	Livebirth	Discordant with adverse outcome
7	Amniocentesis: Normal karyotype	None reported	Term livebirth	Discordant
8^e^	Amniocentesis: Normal karyotype Postnatal placenta: Normal karyotype	None reported	Term livebirth	Discordant
9^f^	Amniocentesis: Karyotype 46,XY, add (20)p13 [2]/46,XY [14]	None reported	Term livebirth	Discordant^f^
10	No diagnostic testing	Spontaneous fetal demise	Fetal demise at 13 weeks	Adverse outcome, no testing

N/a, not applicable

^a^Cases 1–8 could possibly represent confined placental mosaicism. Case 9, although discordant, could likely be explained by the mosaic partial duplication being interpreted as a trisomy 20 by the assay. Case 10 could possibly represent confined placental mosaicism or full or mosaic fetal trisomy 20 as the explanation for the fetal demise

^b^Features consistent with prolonged oligohydramnios, bilateral small kidneys, small bladder, normal ureters, bilateral small lungs, abnormal horizontal sulcus in occipital lobes of brain, small areas of haemorrhage and possible haemosiderin deposition in the brain, possible fibrin thrombus 19 weeks gestation

^c^Emergency preterm birth (C-section) due to cord prolapse. Eight-week stay in the neonatal intensive care unit

^d^Spontaneous preterm birth < 37 weeks

^e^Postnatal testing carried out on placental tissue, 6 biopsy samples all 46, XY

^f^Baby required breathing support initially at birth. Jaundice due to ABO incompatibility, doing well otherwise. Although listed as discordant, it is likely that the mosaic partial duplication (duplication at 20p13) observed was related to the high-risk NIPT result for a trisomy 20

**Fig. 1:**
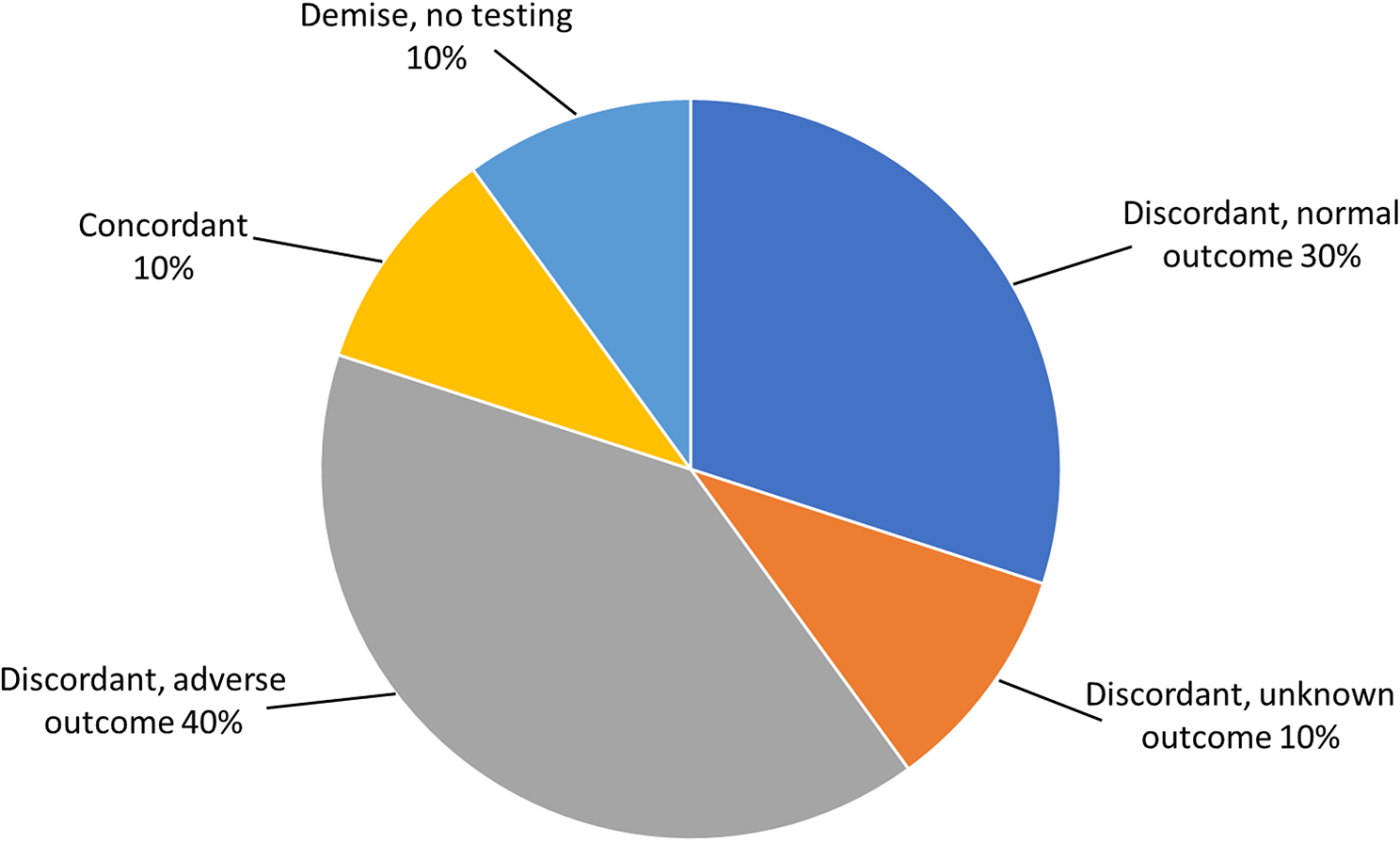
Distribution of outcomes for genome-wide cfDNA screen-positive trisomy 20 cases

Figure 1 shows the distribution of outcomes for all ten cases. Adverse pregnancy outcomes were seen in 50% (5/10), which could suggest the presence of underlying CPM or a mosaic/full fetal trisomy 20, especially for the spontaneous fetal demise case (Table 2). However, none of the cases with adverse outcomes had placental testing. Adverse pregnancy complications observed in these patients included preeclampsia, fetal growth restriction, spontaneous preterm birth, and fetal demise. Of the seven known cases that resulted in a liveborn, birth weights were available for five; none of these cases experienced a low birth weight.

## Discussion

In this study we describe diagnostic and obstetric outcomes for a small cohort of patients who screened positive for presence of trisomy 20 following genome-wide cfDNA screening. Our case series observed a range of outcomes for these 10 cases, from fetal demise in a case without diagnostic testing, to normal, term, live-births in discordant cases (normal amniocentesis result). It is possible that the case that ended in a fetal demise at 13 weeks could have been due to the presence of confined placental mosaicism or full or mosaic fetal trisomy 20, as fetal trisomy 20 often results in an early miscarriage. However, to our knowledge, testing of the products of conception was not carried out in this case. Four other cases that were found to be discordant following diagnostic testing with amniocentesis also had adverse outcomes. While this may be due to underlying CPM, none of these cases had placental testing. Indeed, the other discordant cases without adverse outcomes may also have had underlying CPM. Placental studies in clinical settings are challenging and valuable information regarding CPM associated with RAAs is often not available. A recent study noted that over half of RAA cases with follow-up that were found to be false positives based on fetal testing had confirmed CPM based on placental or chorionic villus biopsy [18]. Confined placental mosaicism has been shown to be associated with a range of pregnancy and birth complications including fetal growth restriction, preterm birth, structural fetal anomalies, and preeclampsia [12, 13, 37, 38]. In a recent study from the TRIDENT group in the Netherlands, CPM trisomy 20 cases were found to be significantly associated with preeclampsia and with an onset of labor by planned caesarean Sect. [13]. Although out study cohort is limited by small sample size, our findings are similar to those seen in previous studies [13, 39].

Studies have shown a range of positive predictive values (PPVs) for RAAs detected by cfDNA screening, with a recent publication noting a pooled PPV of 11.46% in the detection of rare autosomal trisomies based on a meta-analysis of 31 studies [40]. However, most studies base their PPV on concordance with fetal diagnostic testing only, and not placental testing. Therefore, the PPV may in fact be much higher for RAA cases. Unfortunately, a lack of comprehensive diagnostic testing prohibits the calculation of PPV for our study cohort. However, if we consider the possibility that the four cases with adverse pregnancy outcomes but no placental testing may have had CPM of trisomy 20, and that the fetal demise case was a fetal trisomy 20 case, then our study PPV could have been as high as 50% (5/10). In addition, if we also view the case with a mosaic partial duplication (duplication at 20p13) as concordant with the cfDNA result, then the study PPV could have been as high as 60% (6/10).

Given the adverse pregnancy and birth outcomes that can occur with RAAs, some publications have suggested tailored perinatal management for patients who screen positive for presence of a RAA [12, 13]. This could include detailed ultrasound scans, increased monitoring for complications such as fetal growth restriction, and confirmatory diagnostic testing. It has also been suggested that confirmatory testing using CVS may be preferable over amniocentesis for a trisomy 20 result on cfDNA screening as this trisomy is usually involved in CPM type I, i.e., presence of the aneuploidy in the cytotrophoblast only [41]. UPD testing should also be considered for patients with a screen-positive result for trisomy 20 [30], given the potential for a trisomic rescue resulting in a mosaic placenta and euploid fetus. Unfortunately, in this cohort of cases, UPD testing was only carried out in one case. As is the case with all cfDNA screening, appropriate comprehensive pre-and post-test counselling of all patients is needed.

In conclusion, genome-wide cfDNA screening allows screening for additional chromosomal aneuploidies beyond the common trisomies. As the uptake of genome-wide cfDNA screening increases, more data regarding clinical outcomes of RAAs, including chromosome-specific data, will be useful for patient counseling. Diagnostic testing is recommended in the event of any screen-positive cfDNA result [5–7]; placental testing should be considered more systematically for screen-positive cases. In addition, in the event of a screen-positive result involving an imprinted chromosome, UPD testing should also be considered. Based on our series, the fetal outcome for genome-wide cfDNA screen-positive cases for trisomy 20 is encouraging, but pregnancies may be at increased risk for adverse obstetric outcomes and may benefit from additional surveillance. Given that this cohort was comprised mostly of patients of advanced maternal age, further studies are needed to see if similar outcomes are observed in an average-risk obstetric population.

## Data Availability

The datasets generated and/or analyzed during the current study are not publicly available due to patient privacy concerns as well as ethical restrictions but are available from the corresponding author on reasonable request.

## Abbreviations

cfDNA: cell-free DNA
CPM: confined placental mosaicism
RAA: rare autosomal aneuploidy
UPD: uniparental disomy

## References

[1] Rose NC, Barrie ES, Malinowski J, Jenkins GP, McClain MR, LaGrave D (2022). Systematic evidence-based review: the application of noninvasive prenatal screening using cell-free DNA in general-risk pregnancies. Genet Med.

[2] Gil MM, Accurti V, Santacruz B, Plana MN, Nicolaides KH (2017). Analysis of cell-free DNA in maternal blood in screening for aneuploidies: updated meta-analysis. Ultrasound Obstet Gynecol.

[3] Badeau M, Lindsay C, Blais J, Nshimyumukiza L, Takwoingi Y, Langlois S (2017). Genomics-based non-invasive prenatal testing for detection of fetal chromosomal aneuploidy in pregnant women. Cochrane Database Syst Rev.

[4] Mackie FL, Hemming K, Allen S, Morris RK, Kilby MD (2017). The accuracy of cell-free fetal DNA-based non-invasive prenatal testing in singleton pregnancies: a systematic review and bivariate meta-analysis. BJOG.

[5] Hui L, Ellis K, Mayen D, Pertile MD, Reimers R, Sun L (2023). Position statement from the International Society for Prenatal Diagnosis on the use of non-invasive prenatal testing for the detection of fetal chromosomal conditions in singleton pregnancies. Prenat Diagn.

[6] Dungan JS, Klugman S, Darilek S, Malinowski J, Akkari YMN, Monaghan KG (2022). Noninvasive prenatal screening (NIPS) for fetal chromosome abnormalities in a general-risk population: an evidence-based clinical guideline of the American College of Medical Genetics and Genomics (ACMG). Genet Med.

[7] Screening for Fetal Chromosomal Abnormalities (2020). ACOG Practice Bulletin, Number 226. Obstet Gynecol.

[8] Audibert F, De Bie I, Johnson JA, Okun N, Wilson RD, Armour C (2017). 348-Joint SOGC-CCMG Guideline: update on prenatal screening for fetal aneuploidy, fetal anomalies, and adverse pregnancy outcomes. Journal of obstetrics and gynaecology Canada: JOGC = Journal D’obstetrique et gynecologie du Canada. : JOGC.

[9] Dondorp W, de Wert G, Bombard Y, Bianchi DW, Bergmann C, Borry P (2015). Non-invasive prenatal testing for aneuploidy and beyond: challenges of responsible innovation in prenatal screening. Eur J Hum Genet.

[10] De Falco L, Savarese G, Savarese P, Petrillo N, Ianniello M, Ruggiero R (2023). Clinical experience with genome-wide noninvasive prenatal screening in a large cohort of twin pregnancies. Genes.

[11] Zhang M, Tang J, Li J, Wang C, Wei R, Fang Y (2023). Value of noninvasive prenatal testing in the detection of rare fetal autosomal abnormalities. Eur J Obstet Gynecol Reprod Biol.

[12] Mossfield T, Soster E, Menezes M, Agenbag G, Dubois M-L, Gekas J, et al. Multisite assessment of the impact of cell-free DNA-based screening for rare autosomal aneuploidies on pregnancy management and outcomes. Front Genet. 2022;13. 10.3389/fgene.2022.975987. 10.3389/fgene.2022.975987 PMC9465083 36105088

[13] van Prooyen Schuurman L, Sistermans EA, Van Opstal D, Henneman L, Bekker MN, Bax CJ (2022). Clinical impact of additional findings detected by genome-wide non-invasive prenatal testing: follow-up results of the TRIDENT-2 study. Am J Hum Genet.

[14] Harasim T, Neuhann T, Behnecke A, Stampfer M, Holinski-Feder E, Abicht A. Initial clinical experience with NIPT for rare autosomal aneuploidies and large Copy Number variations. J Clin Med. 2022;11(2). 10.3390/jcm11020372. 10.3390/jcm11020372 PMC8777675 35054066

[15] Hu T, Wang J, Zhu Q, Zhang Z, Hu R, Xiao L (2022). Clinical experience of noninvasive prenatal testing for rare chromosome abnormalities in singleton pregnancies. Front Genet.

[16] Xue H, Yu A, Lin M, Chen X, Guo Q, Xu L (2022). Efficiency of expanded noninvasive prenatal testing in the detection of fetal subchromosomal microdeletion and microduplication in a cohort of 31,256 single pregnancies. Sci Rep.

[17] Wang W, Lu F, Zhang B, Zhou Q, Chen Y, Yu B (2022). Clinical evaluation of non-invasive prenatal screening for the detection of fetal genome-wide copy number variants. Orphanet J Rare Dis.

[18] Van Den Bogaert K, Lannoo L, Brison N, Gatinois V, Baetens M, Blaumeiser B (2021). Outcome of publicly funded nationwide first-tier noninvasive prenatal screening. Genet Sci.

[19] Soster E, Boomer T, Hicks S, Caldwell S, Dyr B, Chibuk J (2021). Three years of clinical experience with a genome-wide cfDNA screening test for aneuploidies and copy-number variants. Genet Med.

[20] Rafalko J, Soster E, Caldwell S, Almasri E, Westover T, Weinblatt V (2021). Genome-wide cell-free DNA screening: a focus on copy-number variants. Genet Sci.

[21] van der Meij KRM, Sistermans EA, Macville MVE, Stevens SJC, Bax CJ, Bekker MN (2019). TRIDENT-2: national implementation of genome-wide non-invasive prenatal testing as a first-tier screening test in the Netherlands. Am J Hum Genet.

[22] Liang D, Cram DS, Tan H, Linpeng S, Liu Y, Sun H (2019). Clinical utility of noninvasive prenatal screening for expanded chromosome disease syndromes. Genet Med.

[23] Van Opstal D, van Maarle MC, Lichtenbelt K, Weiss MM, Schuring-Blom H, Bhola SL (2018). Origin and clinical relevance of chromosomal aberrations other than the common trisomies detected by genome-wide NIPS: results of the TRIDENT study. Genet Med.

[24] Liang D, Lin Y, Qiao F, Li H, Wang Y, Zhang J (2018). Perinatal outcomes following cell-free DNA screening in > 32 000 women: clinical follow-up data from a single tertiary center. Prenat Diagn.

[25] Pescia G, Guex N, Iseli C, Brennan L, Osteras M, Xenarios I (2017). Cell-free DNA testing of an extended range of chromosomal anomalies: clinical experience with 6,388 consecutive cases. Genet Med.

[26] Fiorentino F, Bono S, Pizzuti F, Duca S, Polverari A, Faieta M (2017). The clinical utility of genome-wide non invasive prenatal screening. Prenat Diagn.

[27] Scott F, Bonifacio M, Sandow R, Ellis K, Smet ME, McLennan A (2018). Rare autosomal trisomies: important and not so rare. Prenat Diagn.

[28] Forabosco A, Percesepe A, Santucci S (2009). Incidence of non-age-dependent chromosomal abnormalities: a population-based study on 88965 amniocenteses. Eur J Hum Genetics: EJHG.

[29] Pylyp LY, Spynenko LO, Verhoglyad NV, Mishenko AO, Mykytenko DO, Zukin VD (2018). Chromosomal abnormalities in products of conception of first-trimester miscarriages detected by conventional cytogenetic analysis: a review of 1000 cases. J Assist Reprod Genet.

[30] Del Gaudio D, Shinawi M, Astbury C, Tayeh MK, Deak KL, Raca G (2020). Diagnostic testing for uniparental disomy: a points to consider statement from the American College of Medical Genetics and Genomics (ACMG). Genet Med.

[31] Robinson WP, McGillivray B, Lewis ME, Arbour L, Barrett I, Kalousek DK (2005). Prenatally detected trisomy 20 mosaicism. Prenat Diagn.

[32] Bianca S, Ingegnosi C, Tetto C, Cataliotti A, Ettore G (2005). Prenatally detected trisomy 20 mosaicism and genetic counseling. Prenat Diagn.

[33] Hsu LY, Kaffe S, Perlis TE (1987). Trisomy 20 mosaicism in prenatal diagnosis–a review and update. Prenat Diagn.

[34] Willis MJH, Bird LM, Dell’Aquilla M, Jones MC (2008). Expanding the phenotype of mosaic trisomy 20. Am J Med Genet Part A.

[35] Pertile MD, Flowers N, Vavrek D, Andrews D, Kalista T, Craig A (2021). Performance of a paired-end sequencing-based noninvasive prenatal screening test in the detection of genome-wide fetal chromosomal anomalies. Clin Chem.

[36] Illumina. Jan: VeriSeq NIPT Analysis Software (16 samples) User Guide. https://support.illumina.com.cn/content/dam/illumina-support/documents/documentation/software_documentation/veriseq-nipt-analysis-sw-16/veriseq-nipt-analysis-software-guide-16-1000000012693-05.pdf Accessed 2017.

[37] Eggenhuizen GM, Go A, Koster MPH, Baart EB, Galjaard RJ (2021). Confined placental mosaicism and the association with pregnancy outcome and fetal growth: a review of the literature. Hum Reprod Update.

[38] Eggenhuizen GM, Go ATJI, Sauter Z, Hoffer MJV, Haak MC, Geeven G (2024). The role of confined placental mosaicism in fetal growth restriction: a retrospective cohort study. Prenat Diagn.

[39] Lannoo L, van Straaten K, Breckpot J, Brison N, De Catte L, Dimitriadou E (2022). Rare autosomal trisomies detected by non-invasive prenatal testing: an overview of current knowledge. Eur J Hum Genet.

[40] Acreman ML, Bussolaro S, Raymond YC, Fantasia I, Rolnik DL, Da Silva Costa F (2023). The predictive value of prenatal cell-free DNA testing for rare autosomal trisomies: a systematic review and meta-analysis. Am J Obstet Gynecol.

[41] Van Opstal D, Srebniak MI (2016). Cytogenetic confirmation of a positive NIPT result: evidence-based choice between chorionic villus sampling and amniocentesis depending on chromosome aberration. Expert Rev Mol Diagn.

